# Video game loot boxes are linked to problem gambling: Results of a large-scale survey

**DOI:** 10.1371/journal.pone.0206767

**Published:** 2018-11-21

**Authors:** David Zendle, Paul Cairns

**Affiliations:** 1 Department of Computer Science, York St. John University, York, North Yorkshire, United Kingdom; 2 Department of Computer Science, University of York, York, North Yorkshire, United Kingdom; Deakin University, AUSTRALIA

## Abstract

Loot boxes are items in video games that can be paid for with real-world money and contain randomised contents. In recent years, loot boxes have become increasingly common. There is concern in the research community that similarities between loot boxes and gambling may lead to increases in problem gambling amongst gamers. A large-scale survey of gamers (n = 7,422) found evidence for a link (η^2^ = 0.054) between the amount that gamers spent on loot boxes and the severity of their problem gambling. This link was stronger than a link between problem gambling and buying other in-game items with real-world money (η^2^ = 0.004), suggesting that the gambling-like features of loot boxes are specifically responsible for the observed relationship between problem gambling and spending on loot boxes. It is unclear from this study whether buying loot boxes acts as a gateway to problem gambling, or whether spending large amounts of money on loot boxes appeals more to problem gamblers. However, in either case these results suggest that there may be good reason to regulate loot boxes in games.

## Introduction

Loot boxes are virtual items in video games that contain randomised contents but can be paid for with real-world money. They are available for players to buy in popular games like *Overwatch* (40 million players [1]), *Rocket League* (40 million players [2]), and *Counter-Strike*: *Global Offensive* (Over 25 million players [3]). It is estimated that the total amount of revenue generated by loot boxes this year will be approximately $30 billion [4].

The widespread availability of loot boxes in modern video games has led to questions over whether they should be regulated as a form of gambling. As noted in [5], many of the characteristics of loot boxes are commonly associated with gambling. Both when gambling and when buying loot boxes, individuals stake money on the outcome of a future event, whose result is determined at least partially by chance in the hopes of receiving a valuable reward.

Various regulatory organisations have therefore had to recently decide whether they consider loot boxes to legally constitute a form of gambling. This has resulted in a broad spread of decisions. Earlier this year the Belgium Gambling Commission ruled that some loot boxes were in violation of national gambling legislation [6]. More specifically, they ruled that any loot box that can be paid for with real-world money constituted a form of gambling and have ordered that they be removed from video games in Belgium[7]. The Netherlands has similarly ruled that some loot boxes are a form of gambling. However, in contrast to Belgium, they have classified any loot boxes whose contents can be redeemed for real-world money as a form of gambling [8]. Contrastingly, France’s online gambling authority ARJEL have ruled that all loot boxes do not legally constitute a form of gambling as there is no financial value to items that can be won in loot boxes [9]. Controversy over the legal status of loot boxes seems set to continue for the foreseeable future, with bills proposed in recent months in both Washington and Hawaii to regulate games that contain loot boxes [10] [11]. A deeper overview of the various legal issues surrounding loot boxes is presented in [12].

Connected to legal arguments about the status of loot boxes are questions about the effects of loot boxes on gamers. More specifically, there is concern in the academic community that similarities between loot boxes and gambling may lead to problem gambling amongst gamers. Problem gambling can be defined as a pattern of gambling activity which is so extreme that it causes an individual to have problems in their personal, family, and vocational life [13]. These issues range from domestic abuse [14] and intimate partner violence [15] to involvement in illegal activities [16], increased medical costs [17], and suicidality [18]. Problem gambling is typically described as being both excessive and involuntary.

Problem gambling is thought to often be caused by individuals being conditioned by the arousing features of gambling to the point that their need for the excitement of gambling becomes harmful both to themselves and to others [19]. There is reason to believe that such conditioning may occur because of loot box use. In [20], Drummond and Sauer analysed 22 games which featured loot boxes in order to determine if these games had characteristics of gambling that are necessary for such conditioning, and could therefore form a gateway for gamers to become problem gamblers. Their analysis concluded that “in the way they encourage and sustain user engagement, loot-box systems share important structural and psychological similarities with gambling”. They recommended regulation of some specific forms of loot boxes in games, lest they create a “ripe breeding ground” for problem gambling amongst gamers.

Conversely, it may be the case that similarities between loot boxes and gambling are the root of a different relationship between problem gambling and loot box use. As noted above, problem gambling is characterised by an excessive and harmful involvement with gambling activities. There are key similarities between loot boxes and gambling. These similarities may cause individuals who are already problem gamblers to spend large amounts of money on buying loot boxes in games, just as they would spend large amounts of money on other forms of gambling. If loot boxes are attractive to those with problem gambling behaviours, they pose a serious moral question for the games companies who profit from them.

However, criticism of loot boxes has been roundly rebuffed by representatives of the games industry, with the ESRB recently claiming that there was insufficient evidence to state that loot boxes had negative consequences for gamers. They instead declared that “we do not consider loot boxes to be gambling for various reasons … loot boxes are more comparable to baseball cards, where there is an element of surprise and you always get something.” [21].

The position that there is currently no strong evidence of a link between loot box use and problem gambling is tenable. Loot boxes, whilst extremely widespread, are a relatively recent phenomenon. In [22], Macey and Hamari found a potentially important link between problem gambling and loot boxes spending amongst eSports spectators. Whilst the authors suggested that due to the composition of their sample their results were not generalisable to the wider population of gamers, they noted “a need for increased attention, from academia and regulators, regarding newly emergent gambling behaviours in contemporary digital culture”. Aside from the research outlined above, there is no current empirical study in existence that examines the relationship between loot boxes and problem gambling. Such work is urgently needed. In a recent editorial to *Addiction* [23], King and Delfabbro called on the community to immediately begin work that investigates whether there are any links between loot box use and gaming-related harm. These concerns about the effects of loot boxes on gamers are echoed by policymakers, with the Australian Senate recently authorising a committee enquiry into the extent to which loot boxes may be harmful to their players [24].

The research that is presented below addresses this lack of research and provides evidence that is of direct relevance to ratings boards and gambling regulators. With reference to spending, it is important to note that some games can also feature loot boxes that are not bought with real-world money. As the primary issue in the regulatory community is around whether loot boxes are gambling, we have focused on loot boxes which require monetary outlay by the players, and the spending associated with these loot boxes. There may be deeper concerns about such ‘unpaid openings’ leading to problematic behaviour more akin to gambling. This is not our immediate concern.

We surveyed a large international sample of gamers (n = 7,422) and measured both how much these individuals spent on loot boxes, and the severity of their problem gambling. By doing so we established both the existence, the size, and the importance of links between purchasing loot boxes and problem gambling.

## Method

### Design

We conducted an online survey with a self-selected sample of gamers aged 18 or older. This survey was available only in English. Participants were recruited via reddit, a popular online bulletin board. The recruitment message stated that we were interested in understanding links between loot boxes and gambling, and that gamers could take part regardless of whether they had previously purchased loot boxes. Participants were not remunerated for their participation. A total of 29 links to the survey were placed on a variety of gaming-related special interest pages (or ‘subreddits’) on this site.

Demographic details about participants were collected, as were quantitative measures of ***problem gambling*, *loot box spending*,** and ***other microtransaction spending*.**

This research was ethically approved by the Cross-School Research Ethics Committee for the Schools of Art, Design & Computer Science of York St. John University. A statement from this internal review board to this effect is available from the authors on request.

***Problem gambling*** was measured using the Problem Gambling Severity Index (PGSI) [25]. This nine-item instrument contains a series of questions about how frequently individuals have engaged in a variety of gambling-related behaviours in the past 12 months (e.g. ‘Have you needed to gamble with larger amounts of money to get the same feeling of excitement?’, ‘Have you borrowed money or sold anything to get money to gamble?’).

Individuals must indicate how frequently they engage in these activities on a four-point scale ranging from ‘Never’ to ‘Almost Always’. These responses are each scored from 0–3, with their sum forming a total score ranging from 0 to 27. The severity of participants’ problem gambling is then classified on the basis of these scores, using the revised scoring system presented in [26]: Individuals who score 0 are classified as ‘non problem gamblers’; those who score 1–4 are classified as ‘low-risk gamblers’; those who score 5–7 are classified as ‘moderate-risk gamblers’; and those who score 8+ are classified as ‘problem gamblers’.

***Loot box spend*** was measured using a series of two questions. Participants were first asked whether they had ever bought a loot box in a video game (Yes/No). If they indicated that they had bought a loot box, they were asked “Approximately how much money in US dollars would you say that you spend on loot boxes each month?”. This was the exact wording of the question, with no date range specified. This question had 13 possible responses: (1) Less than $1; (2) $1-$5; (3) $5-$10; (4) $10-$15; (5) $15-$20; (6) $20-$30; (7) $30-$40; (8); $40-$50; (9) $50-$75; (10) $75-$100; (11) $100-$200; (12) $200-$300; (13) Greater than $300. For the purposes of analysis, those who indicated in the first question that they had never bought a loot box in a game were coded as (0).

***Other in-game microtransaction spend*** was measured to check whether any observed relationship between ***loot box spend*** and ***problem gambling*** was due to the specific features of loot boxes, and not due to individuals who were problem gamblers spending more money in general.

This variable was measured in a similar way to ***loot box spend***. Participants were first asked “Have you ever bought any other item or product in a game using real-world money? (Excluding loot boxes)” (Yes/No). If they indicated that they had bought an item which was not a loot box, they were then asked “Approximately how much money in US dollars would you say that you spend on these items per month? (Excluding loot boxes)”. This was the exact wording of the question, with no date range specified. This question had the same 13 possible responses as the measure of loot box use: (1) Less than $1; (2) $1-$5; (3) $5-$10; (4) $10-$15; (5) $15-$20; (6) $20-$30); (7) $30-$40; (8); $40-$50; (9) $50-$75); (10) $75-$100; (11) $100-$200; (12) $200-$300; (13) Greater than $300. For the purposes of analysis, as with ***loot box spend*,** those who indicated that they had never engaged in in-game microtransactions were coded as (0) No further filter questions were incorporated into this study.

### Participants

14,182 responses were collected in total from gamers. 3173 participants did not give details of their ages and were removed from the study prior to analysis for ethical reasons. 872 participants listed their ages as numbers less than 18, and were removed from the study prior to analysis for ethical reasons. Two participants listed their ages as numbers greater than 120, were deemed non-serious and were removed from analysis. Two participants listed their monthly spend on gambling as greater than $1,000,000, and 9 participants listed their monthly spend on gambling as a negative number. They were deemed non-serious and removed from analysis. 2,702 incomplete responses were removed from the study and not analysed. This left a total of 7,422 responses.

Most participants had engaged in both purchasing loot boxes and buying other in-game items with real-world money. 5793 (78%) of the participants had bought a loot box in a video game, whilst 1629 had not. 6441 (87%) participants had bought an item other than a loot box in a video game using a microtransaction, whilst 981 participants had not.

Most participants, 6,612 (89%), described themselves as male and 694 (9%) as female. Nearly half of the participants (3,589, 48%) were 18–24. 2,066 (27.8%) were aged 25–29; 1,061 (14.3%) were aged 30–34; 444 (6.0%) were aged 35–39; only 262 (3.5%) were in the age groups above 45. Whilst this sample may seem overly skewed towards the presence of young males, it is important to note that it is similar in composition to other studies of gamers in the literature (e.g. [22], [27])

There was no dominant group in terms of annual household income. Incomes ranged from less than $10,000 pa to above $100,000 pa. Most participants were from the US (3290, 44%), UK (572, 8%) and Canada (525, 7%). 382 participants (5%) did not state their nationality. Additionally, there were respondents from 92 other countries.

## Results

**Fig 1:**
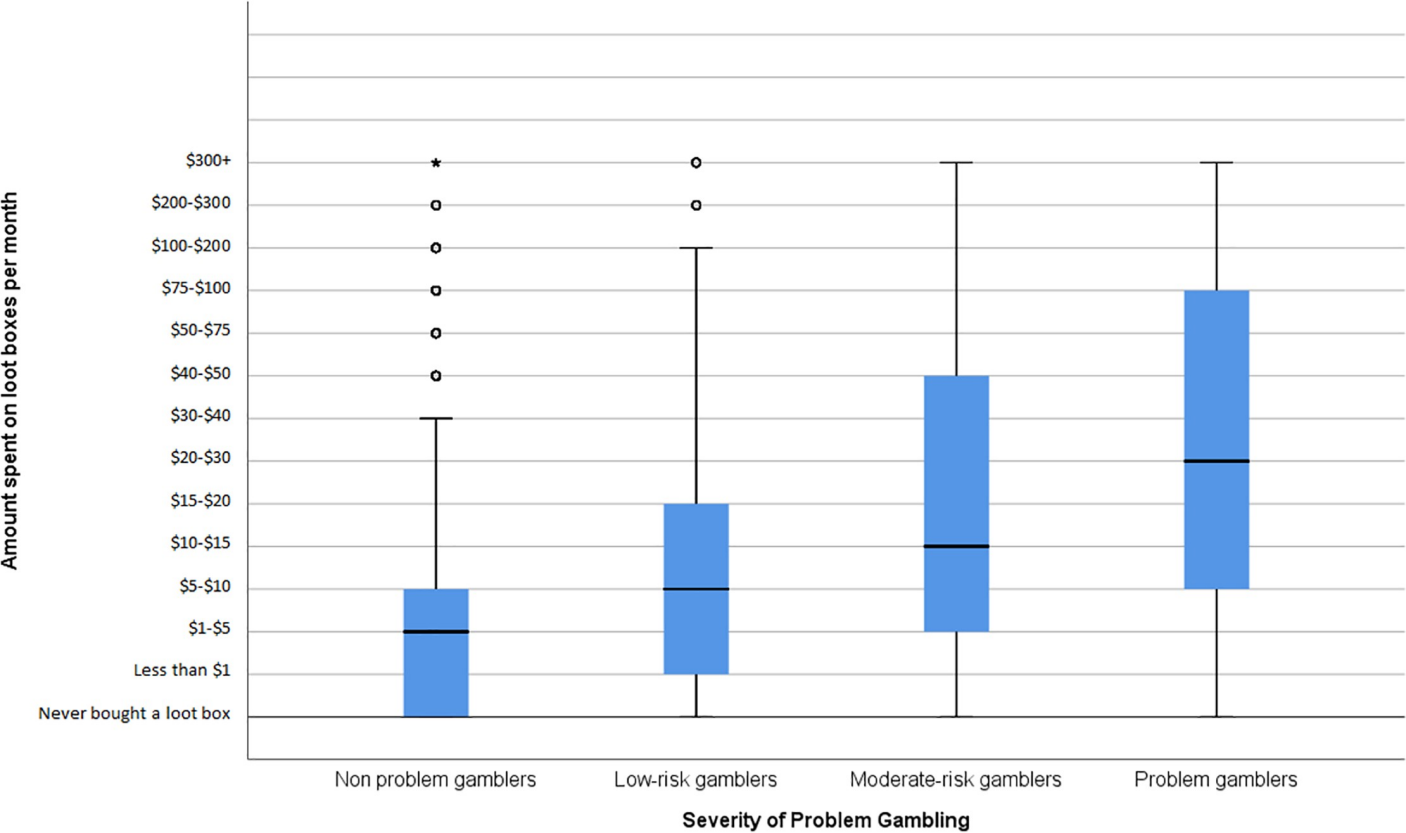
Box-plot of spend on loot boxes, split by severity of problem gambling. Hinges represent 25^th^ and 75^th^ percentiles. Whiskers represent 1.5 times the IQR. Cental line represents the median. Circles represent outliers.

**Fig 2:**
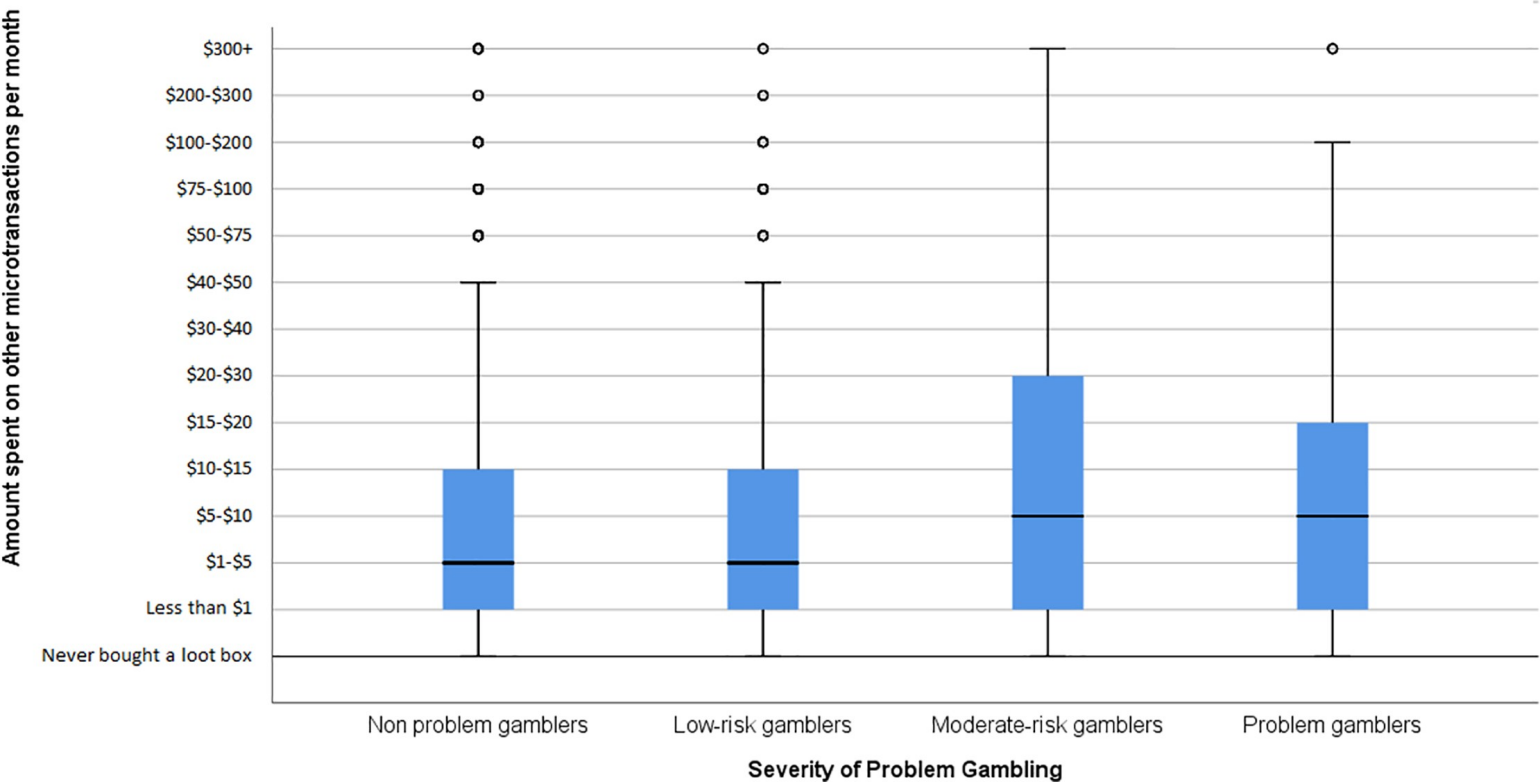
Box-plot of spend on other micro-transactions in games, split by severity of problem gambling Hinges represent 25^th^ and 75^th^ percentiles. Whiskers represent 1.5 times the IQR. Cental line represents the median. Circles represent outliers.

A box plot showing the relationship between loot box spend and problem gambling is presented below as Fig 1. A box plot showing the relationship between other microtransaction spend and problem gambling is presented below as Fig 2. Means and standard deviations for each variable are presented below as Table 1.

**Table 1:** Means and standard deviation of both loot box spending and other microtransaction spending, split by problem gambling severity. Standard deviations in brackets. The spend statistics reported here are mean categories, rather than a conversion into dollar figures. Relevant categories for each mean score are given below each statistic.

	Loot box spend	Other microtransaction spend	N
Non problem gamblers	2.41 (2.57) Category 2: $1-$5 Category 3: $5-$10	2.69 (2.36) Category 2: $1-$5 Category 3: $5-$10	5726
Low-risk gamblers	3.67 (3.12) Category 3: $5-$10 Category 4: $10-$15	3.04 (2.61) Category 3: $5-$10 Category 4: $10-$15	1422
Moderate-risk gamblers	4.96 (3.77) Category 4: $10-$15 Category 5: $15-$20	4.03 (3.38) Category 4: $10-$15 Category 5: $15-$20	170
Problem gamblers	6.47 (4.01) Category 6: $20-$30 Category 7: $30-$40	3.57 (3.54) Category 3: $5-$10 Category 4: $10-$15	104
Total	2.77 (2.84) Category 2: $1-$5 Category 3: $5-$10	2.80 (2.47) Category 2: $1-$5 Category 3: $5-$10	7422

The effects of problem gambling (non problem gamblers, low-risk gamblers, moderate-risk gamblers, problem gamblers) on loot box spend were tested via a Kruskal Wallis H Test. Results indicated that there was a statistically significant effect of problem gambling on loot box spending, χ^2^(3) = 284.255, p<0.001, η^2^ = 0.054.

Pairwise comparisons were then conducted to measure the effects of problem gambling on loot box spending between all groups of problem gamblers via a series of 6 Mann-Whitney U tests. Bonferroni corrections were applied to the results of these tests, lowering the alpha level of the tests to 0.05/6, or 0.008. The results of these comparisons are reported below as Table 2.

**Table 2:** Pairwise comparisons of the effects of problem gambling on loot box spending. Effects that are significant at the p<0.008 level are marked with a *.

Pairwise comparison groups	U	p-value	Cohen’s d
Non problem gamblers vs. low-risk gamblers	3004462	<0.001*	0.368
Non problem gamblers vs. moderate-risk gamblers	282036.5	<0.001*	0.246
Non problem gamblers vs. problem gamblers	119725	<0.001*	0.277
Low-risk gamblers vs. moderate-risk gamblers	97550.5	<0.001*	0.207
Low-risk gamblers vs. problem gamblers	43479.5	<0.001*	0.365
Moderate-risk gamblers vs. problem gamblers	6875	0.002*	0.38

The effects of problem gambling (non problem gamblers, low-risk gamblers, moderate-risk gamblers, problem gamblers) on other microtransaction spend in games were then tested via a Kruskal Wallis H-Test. Results indicated that there was a statistically significant effect of problem gambling on other microtransaction spending, χ^2^(3) = 38.622, p<0.001, η^2^ = 0.004.

Pairwise comparisons were then conducted to measure the effects of problem gambling on other microtransaction spending between all groups of problem gamblers via a series of 6 Mann-Whitney U tests. Bonferroni corrections were applied to the results of these tests, raising the alpha level of the tests to 0.05/6, or 0.008. The results of these comparisons are reported below as Table 3.

**Table 3:** Pairwise comparisons of the effects of problem gambling on other microtransaction spending. Effects that are significant at the p<0.008 level are marked with a *.

Pairwise comparison groups	U	p-value	Cohen’s d
Non problem gamblers vs. low-risk gamblers	3781297.5	<0.001*	0.099
Non problem gamblers vs. moderate-risk gamblers	379105	<0.001*	0.128
Non problem gamblers vs. problem gamblers	267593.5	0.072	0.046
Low-risk gamblers vs. moderate-risk gamblers	102252	0.001*	0.165
Low-risk gamblers vs. problem gamblers	71167.5	0.517	0.033
Moderate-risk gamblers vs. problem gamblers	7965.5	0.166	0.167

## Discussion

The results of this study suggest that there is an important relationship between problem gambling and the use of loot boxes. The more severe that participants’ problem gambling was, the more money they spent on loot boxes. Non problem gamblers spent the least amount of money on loot boxes (mean = 2.41, Category 2 = $1 - $5, Category 3 = $5-$10); low-risk gamblers spent more (mean = 3.67, Category 3 = $5 - $10, Category 4 = $10-$15); moderate-risk gamblers spent yet more (mean = 4.96, Category 4 = $10 - $15, Category 5 = $15-$20); and problem gamblers spent the most of all on loot boxes (mean = 6.47, Category 6 = $20 - $30, Category 7 = $30-$40).

This is not a weak or unimportant relationship. The overall effect of problem gambling on loot box spending was measured at η^2^ = 0.054, indicating that it is of small-to-medium size [28]. Effects of this magnitude commonly bear practical, as well as statistical significance [29]. Indeed, the relationship observed here is stronger than relationship between problem gambling and several common risk factors in the gambling literature. For instance, it is stronger than the relationship between problem gambling and depression (Rho = 0.10, equivalent d = 0.063) and major drug problems (r = 0.12, equivalent d = 0.238) [30]. It is comparable in strength to the relationship between problem gambling and current alcohol dependence (r = 0.25, equivalent to d = 0.516) [31]. If the relationship between loot box spending and problem gambling was of a significantly smaller magnitude than important risk factors in the literature, it would be possible to dismiss the effects of any link between loot box spending and problem gambling as trivial and of little practical importance. However, this is clearly not the case.

Furthermore, the pairwise comparisons that were conducted to clarify the effects of the initial analysis paint an even starker picture of the relationship between problem gambling and loot box use. They show that every increase in classification of problem gambling severity amongst gamers comes with an associated increase in loot box spending.

The strength of the relationship observed here was specific to loot boxes. It did not apply to other kinds of spending in video games. Whilst a significant relationship was observed between problem gambling and other microtransaction spend in games, it was much weaker (η^2^ = 0.004) than the relationship between problem gambling and loot boxes. In other words, increases in problem gambling corresponded to increases in the amount spent on other microtransactions in games. However, these increases were much smaller than the increases in spending that were associated with loot box use: For example, the difference in spending on microtransactions between non problem gamblers and problem gamblers was of d = 0.046 –more than 5 times smaller than the effect of problem gambling on spending on loot boxes between these groups.

### Further work

The effects that are reported here are of practical significance and bear important weight for policy-makers. However, this is amongst the first research that looks at relationships between problem gambling and loot boxes, and it is important to acknowledge the limitations of this study and point to further work that is necessary to build on these findings.

The primary limitation of this study is its correlational nature. As described above, it is impossible to understand the direction of the causal relationship between loot box spending and problem gambling from the results reported here. To determine which way this relationship flows, significant further longitudinal and experimental work is needed.

An additional limitation relates to the unblinded nature of the sample who took part in this research. Whilst participants were not aware of the specific aims of this study, they did know that it related to both loot boxes and gambling. This knowledge may have influenced their reporting. For example, they may have reported either greater or lesser levels of gambling and/or loot box spending due to the belief that these things were expected by the researchers. Further work is needed to determine whether the size of the relationships observed here replicates to situations where participants are entirely unaware of the study’s aims.

Furthermore, both the self-selected nature of the sample and the fact that participants were recruited from the online bulletin board ‘reddit’ may also limit the generalisability of these findings. For example, participants who were particularly concerned about how their loot box use might be affecting their gambling might have been disproportionately represented in our sample, influencing our results. Similarly, the kind of gamers who are likely to be recruited via reddit may not be representative of gamers. However, it is important to note that the data suggest that both of these situations seem unlikely: Not only (as noted in the Method) was the composition of our sample similar to other studies in the field in terms of age and gender, but the proportion of problem gamblers that were observed in the study is similar (at approximately 0.1% of the sample) to estimations of the general prevalence of problem gambling, which place problem gambling as occurring at between 0.4% and 1.3% of the general population [26]. However, further work is still needed to confirm that these effects replicate across other groups of gamers.

An additional limitation of this study was that we asked participants to indicate their level of spend on an ordinal set of categories. These categories are not evenly spaced because of the potential for a long-tail in this sort of data. This form of data collection gives an inherent degree of measurement error. However, it is frequently seen in studies of this sort (e.g. [32–34]). It is important to note that the reduction to a limited set of ordinal values acts to restrict any differences seen. The effects in terms of actual spend could in fact be substantially greater than those observed here. This ordinal scale is therefore conservative as a way to estimate effects in this domain. Further work is needed to determine whether measurement of spend in absolute dollar values captures a larger effect size from the population than the method used here.

Finally, given the limitation of this study to consider only paid loot box opening and not consider relationships of problem gambling with unpaid loot boxes, it would be very important to examine these separately and so extend our findings. Furthermore, whilst we defined loot boxes in the context of this research as items that are paid for with real-world money, some participants of the study who only used unpaid openings may have still taken part and been merged into the ‘less than $1’ spending option. Further work that examines the specific relationship between unpaid openings and problem gambling in separation from paid openings may lead to a better understanding of the pathways between problem gambling and loot box behaviours if the relation found here extends to this wider class of loot boxes.

## Conclusions

This research provides empirical evidence of a relationship between loot box use and problem gambling. The relationship seen here was neither small, nor trivial. It was stronger than previously observed relationships between problem gambling and factors like alcohol abuse, drug use, and depression. Indeed, sub-group analyses revealed that an individual’s classification as either a non problem gambler or a problem gambler accounted for 37.7% of the variance in how much they spent on loot boxes.

These results may confirm the existence of the causal relationship between buying loot boxes and problem gambling that was theoretically proposed in [20]. Due to the formal features that loot boxes share with other forms of gambling, they may well be acting as a ‘gateway’ to problem gambling amongst gamers. Hence, the more gamers spend on loot boxes, the more severe their problem gambling becomes.

However, it is important to note that this is not the only causal relationship which fits the data. It may be the case that individuals who are already problem gamblers instead tend to spend more on loot boxes. There are good reasons why this might be the case. Loot boxes share key similarities with other kinds of gambling. Since problem gambling is characterised by uncontrollable and disordered spending on gambling activities, this lack of control and excess in spending may apply to loot boxes too. Hence, the more severe a gamer’s problem gambling, the more they spend on loot boxes. If this is the case, then loot boxes in digital games would be providing less of a ‘breeding ground’ for problem gambling. They would instead be providing another outlet for individuals who are already problem gamblers to engage in harmful and excessive gambling-related behaviour.

Due to the correlational nature of this research, it is impossible to tease apart whether we are seeing a situation in which spending on loot boxes leads to problem gambling, or whether we are seeing a situation in which problem gambling leads to spending on loot boxes. It may, indeed be the case that both directions of causality are true: Problem gamblers spend more on loot boxes, whilst buying loot boxes simultaneously leads to increases in problem gambling amongst gamers.

However, regardless of which of these outcomes is the case, this research bears an important message when it comes to the regulation of loot boxes within the gaming industry. Industry analysts predict that loot boxes will drive a large proportion of the revenue generated in the $230 billion [35] video game economy by 2022. Gamers are already projected to spend approximately $30 billion on loot boxes this year alone, with this figure rising to $50 billion over the next four years [4]. It may be the case that this spending is leading to problem gambling. It may be that this level of spending is driven by pre-existing problem gambling amongst gamers. Further experimental and longitudinal work is required to establish the direction of this causal relationship. However, in either case, this research provides industry bodies such as the ESRB with crucial evidence to use when determining whether there is still insufficient evidence of links between problem gambling and loot box use.

This study shows a relationship between loot box spending and problem gambling. We believe that the strength and direction of this relationship indicates that regulation of loot boxes is appropriate and necessary. For example, as suggested by [20], ratings agencies such as the ESRB and PEGI may wish to incorporate additional parental advisories into games that feature loot boxes, and may consider restricting access to games that feature loot boxes to players of legal gambling age.

Furthermore, we believe that the strength of the relationship that was observed here between problem gambling and loot box spending suggests that important gambling-related harm is experienced by users of loot boxes. We strongly recommend that relevant national and federal regulatory authorities consider restricting access to loot boxes as if they were a form of gambling.

Whether loot boxes fulfil the technical requirements to be classified as gambling is a legal matter that will vary from territory to territory and from country to country. However, the evidence presented here clearly shows that there is a very real relationship between loot box spending and problem gambling. It is our opinion that this relationship remains serious and potentially dangerous regardless of whether loot boxes are technically considered a form of gambling or not.

## Supporting information

S1 File Survey results in SPSS SAV format (PLOS ONE Data Loot Boxes.sav). (SAV)

S2 File Survey results in SPSS Excel format (PLOS ONE Data Loot Boxes.xlsx). (XLSX)
