# Correction: Video game loot boxes are linked to problem gambling: Results of a large-scale survey

**DOI:** 10.1371/journal.pone.0214167

**Published:** 2019-03-14

**Authors:** David Zendle, Paul Cairns

In the Conclusions section, there is an error in the last sentence of the first paragraph. It refers to the results of a parametric statistical test which appeared in the preprint, but was not used in the published manuscript. Therefore, the following sentence is not applicable: Indeed, sub-group analyses revealed that an individual’s classification as either a non problem gambler or a problem gambler accounted for 37.7% of the variance in how much they spent on loot boxes.
